# A Peircean framework for explainable and human-centred artificial intelligence

**DOI:** 10.3389/frai.2026.1962744

**Published:** 2026-09-29

**Authors:** Majeda Ahmad Omar, Ahmad Abdalla AlAhmad

**Affiliations:** 1 Department of Philosophy, School of Arts, The University of Jordan, Amman, Jordan; 2 Information Technology Center, The University of Jordan, Amman, Jordan

**Keywords:** Charles S. Peirce, explainable AI, human-centred AI, pragmaticism, semiotics, trustworthy AI

## Abstract

This article develops a philosophical-conceptual framework for rethinking Explainable Artificial Intelligence (XAI) and Human-Centred Artificial Intelligence (HCAI) through the pragmatic semiotics of Charles Sanders Peirce. Rather than treating explainability solely as algorithmic transparency, the article conceptualizes it as a socially situated epistemic process through which AI-generated outputs are interpreted, evaluated, contested, and revised within human practices of inquiry. Drawing on Peirce’s concepts of semiosis, abduction, inquiry, fallibilism, pragmaticism, and the community of inquiry, the framework distinguishes between computational processes performed by AI systems and the human and institutional practices through which their outputs acquire epistemic and practical significance. AI outputs may function as signs and may generate hypotheses that are functionally analogous to abductive possibilities; however, interpretation, evidential assessment, justification, ethical judgment, and responsibility remain situated within human communities of inquiry. The framework further translates Peircean concepts into practical principles for XAI, including contextual interpretation, uncertainty communication, contestability, human review, stakeholder participation, evidence-based revision, and continuous evaluation. A worked example involving generative AI illustrates how an AI-generated explanation can move through interpretation, questioning, evidence checking, contestation, and revision rather than being accepted as a final epistemic product. The article therefore positions Peircean pragmatism not as a replacement for technical XAI methods but as a philosophical and normative framework that complements them by clarifying the epistemological conditions of meaningful explanation, trustworthy human oversight, and responsible AI governance.

## 1 Introduction

Recent advances in artificial intelligence (AI) have transformed intelligent systems from computational tools into socio-technical infrastructures that increasingly shape decision-making, knowledge production, education, healthcare, governance, and everyday life. As AI becomes embedded in critical social practices, the central philosophical challenge is no longer whether these systems can perform complex computational tasks, but how their outputs should be understood, interpreted, and evaluated as forms of knowledge. Consequently, contemporary debates have shifted beyond algorithmic performance toward broader questions concerning explanation, meaning, responsibility, trust, and human agency. AI should therefore be understood not merely as a technological innovation but as a socio-technical transformation with profound ethical, legal, and epistemological implications (Qian et al., 2024).

One of the most significant challenges arising from this transformation concerns the nature of explainability. While contemporary research on Explainable Artificial Intelligence (XAI) has produced numerous technical methods for improving the transparency of AI systems, philosophical disagreement remains regarding what constitutes a genuine explanation and how AI-generated outputs acquire epistemic significance for human users. These concerns have also motivated the development of AI governance frameworks that emphasize transparency, accountability, human oversight, and social responsibility in the design and deployment of intelligent systems (Emery-Xu et al., 2025). Nevertheless, existing approaches frequently conceptualize explainability as a technical property of algorithms rather than as an interpretive process through which meaning is constructed and evaluated.

This philosophical limitation suggests the need for an alternative conceptual framework capable of explaining AI not simply as computational information processing but as a process of interpretation and knowledge formation. Among the philosophical traditions capable of addressing this challenge, Charles Sanders Peirce’s pragmaticism offers particularly valuable conceptual resources. Unlike approaches that regard knowledge as a fixed product of rational cognition, Peirce understood knowledge as a dynamic and self-correcting process emerging through semiosis, inquiry, abductive reasoning, and the continuous revision of beliefs (Peirce, 1878). His integration of semiotics, logic, and scientific inquiry provides a coherent epistemological framework for explaining how meaning is generated, hypotheses are formed, and knowledge develops through interpretation and communal criticism (Burks, 1996; Peirce, 1998, p. 358). Recent philosophical scholarship has likewise highlighted the renewed relevance of Peirce’s philosophy for contemporary AI by interpreting intelligent systems as participants in processes of meaning formation, inquiry, and knowledge development rather than as purely computational mechanisms (Zanette, 2025).

Despite the growing literature on explainable, trustworthy, and human-centred AI, the literature reviewed here has paid comparatively limited attention to Peirce’s pragmatic semiotics as a philosophical framework for understanding explainability as a semiotic and interpretive process rather than merely a technical feature of intelligent systems. Most existing discussions focus on algorithmic transparency, governance, or ethical principles, while giving comparatively limited attention to the epistemological conditions through which AI-generated outputs become meaningful, interpretable, and socially accountable.

Building on this gap, the present study develops a philosophical-conceptual and normative framework that reconstructs Peirce’s pragmatic semiotics in relation to contemporary Explainable Artificial Intelligence (XAI) and Human-Centred Artificial Intelligence (HCAI). The study does not propose a new computational explanation technique or empirically evaluate an AI system. Instead, it asks how explanations acquire meaning and epistemic significance within human practices of interpretation and inquiry. By integrating semiosis, abduction, inquiry, fallibilism, pragmaticism, and the community of inquiry, the study argues that explainability is an iterative process in which AI-generated outputs are interpreted, evaluated against evidence and context, contested when necessary, and revised through human and institutional inquiry. The framework therefore complements technical XAI by providing philosophical and normative criteria for meaningful explanation, human oversight, contestability, and responsible AI governance.

The remainder of the article presents the method, develops the Peircean theoretical foundations, maps them onto XAI design and governance, applies the framework to a generative-AI example, and discusses its implications for explainability and responsible AI.

### 1.1 Research question

This study addresses the following research questions:

How can Peirce’s pragmatic semiotics be reconstructed as a philosophical framework for explainable and human-centred artificial intelligence, and what new epistemological insights does this framework offer for understanding interpretation, knowledge production, and responsible AI governance?

### 1.2 Research objectives

This study aims to:

1. Develop Peirce’s pragmatic semiotics as a philosophical framework for explainable and human-centred artificial intelligence, integrating semiosis, abduction, inquiry, fallibilism, pragmaticism, and the community of inquiry.
2. Reconceptualize Explainable Artificial Intelligence (XAI) as an epistemological and interpretive process in which AI-generated explanations acquire meaning through human interpretation and inquiry rather than algorithmic transparency alone.
3. Demonstrate how Peirce’s philosophy enriches contemporary debates on trustworthy AI and AI governance by providing an interpretive framework for understanding knowledge production, accountability, and responsible human oversight.

## 2 Method and philosophical reconstruction

This study adopts a philosophical-conceptual methodology combining descriptive-analytical interpretation, comparative philosophical analysis, and conceptual reconstruction. The analysis proceeds by examining selected concepts in Peirce’s pragmatic semiotics, particularly semiosis, abduction, inquiry, fallibilism, pragmaticism, and the community of inquiry, and then reconstructing their epistemological significance in relation to contemporary Explainable Artificial Intelligence (XAI) and Human-Centred Artificial Intelligence (HCAI).

The analysis is not an empirical investigation of AI performance and does not involve statistical testing, user experiments, or comparative evaluation of explanation techniques. Instead, it follows a conceptual and philosophical procedure. First, the relevant Peircean concepts are interpreted within their philosophical context. Second, their epistemological functions are distinguished from computational processes performed by contemporary AI systems. Third, these functions are compared with current approaches to explainability, human-centred AI, and contestable AI. Finally, the resulting relationships are synthesized into a normative framework specifying implications for explanation, human interpretation, evidence-based evaluation, contestability, oversight, and governance.

The comparative component is used primarily to clarify conceptual differences and complementarities rather than to rank competing approaches. In particular, the analysis distinguishes Peircean inquiry from computational adaptation, abductive hypothesis formation from statistical output generation, and human interpretation from automated information processing. The resulting framework is therefore a philosophical reconstruction: it uses Peircean concepts to clarify the epistemological conditions under which AI-generated outputs can become meaningful, evaluable, contestable, and practically useful within human and institutional practices.

## 3 Theoretical framework

### 3.1 Why Peirce for contemporary AI

AI has evolved from a computational technology primarily concerned with automation and data processing into a complex socio-technical system that increasingly influences knowledge production, decision-making, communication, and social interaction. This transformation can also be understood historically. Early computational conceptions of intelligence were grounded in formal models of computation, particularly Turing’s foundational work on computability, which established the theoretical basis for modern computer science before later developments expanded AI toward cognitive, interpretive, and socio-technical perspectives (Turing, 1936). As AI applications become embedded in fields such as healthcare, education, governance, and scientific research, evaluating intelligent systems requires more than assessing computational efficiency or predictive accuracy. It also requires philosophical frameworks capable of explaining how AI participates in processes of meaning construction, interpretation, and knowledge formation within broader social contexts.

Charles Sanders Peirce’s philosophy provides a particularly useful framework for this problem because it treats knowledge, meaning, and inquiry as interconnected processes rather than as static properties of representations (Pietarinen, 2006). His pragmatic semiotics links the interpretation of signs with the formation and testing of hypotheses, while his conception of inquiry emphasizes the revisability of beliefs through experience, criticism, and communal investigation. This combination is especially relevant to contemporary AI because AI-generated outputs increasingly function as inputs to human processes of interpretation and decision-making. The philosophical question is therefore not simply whether an AI system produces accurate representations, but how those representations become meaningful, evidentially assessable, and practically relevant within particular contexts of use.

Peirce’s relevance should not, however, be understood as evidence that contemporary AI systems instantiate Peircean cognition. His philosophy is valuable precisely because it provides conceptual distinctions that can prevent such an inference. AI systems can generate predictions, classifications, texts, and candidate explanations, but these outputs do not by themselves establish their own meaning, justification, or practical validity. The Peircean perspective instead directs attention to the broader process in which outputs are interpreted, hypotheses are considered, evidence is examined, and beliefs are revised. AI can therefore be situated within inquiry without being identified with the human or communal practices through which inquiry acquires epistemic significance.

### 3.2 The development of Peirce’s pragmaticism and inquiry

Peirce’s pragmaticism developed as an attempt to clarify the relationship among meaning, inquiry, experience, and knowledge. In “How to Make Our Ideas Clear,” he formulated the Pragmatic Maxim as a method for clarifying concepts through their conceivable practical consequences (Peirce, 1878). Meaning is thus connected to what could follow in experience and inquiry rather than to purely abstract definition.

Peirce later refined this position because he was concerned that pragmatism might be reduced to immediate usefulness. He therefore introduced the term “pragmaticism” to emphasize the logical and epistemological character of his view, linking practical consequences to inquiry, fallibilism, and the progressive clarification of knowledge (Peirce, 1998; Olshewsky, 1983). Pragmaticism should not, therefore, be understood as the claim that whatever is useful is necessarily true. Rather, it connects meaning with consequences that can be investigated within a broader process of inquiry, while concepts, hypotheses, and beliefs remain subject to evidence, criticism, experience, and revision (Hookway, 2000; Haack, 2014). In this respect, pragmaticism forms part of a wider epistemological orientation in which knowledge is understood as an open and self-correcting process rather than as the possession of indubitable foundations.

Peirce’s conception of inquiry can be clarified through its contrast with the Cartesian search for secure foundations. Whereas Cartesian epistemology gives priority to doubt as a route toward indubitable knowledge, Peirce rejects inquiry from a position independent of prior beliefs, signs, and experience. He instead emphasizes socially situated inquiry in which beliefs and hypotheses are exposed to criticism and correction (Haack, 1982; Buczynska-Garewicz, 1994). Recent discussions of Cartesian AI similarly raise questions about artificial thinking and reasoning (Pernagallo, 2026a, 2026b). From a Peircean perspective, however, the key issue is whether AI outputs can be incorporated responsibly into human inquiry without confusing computational generation with epistemic agency.

Inquiry is central to mature Peircean pragmaticism (Peirce, 1931–1958). Knowledge develops through semiosis, abduction, inquiry, and revision: signs require interpretation, unexpected observations can prompt hypotheses, and hypotheses are examined through evidence, experience, and criticism (Hookway, 1997, 2000; Misak, 2004). The resulting epistemology is neither foundationalist nor relativistic: beliefs are fallible but inquiry remains oriented toward correction.

This framework is useful for AI because predictions, classifications, recommendations, and explanations increasingly enter human decision-making. AI can generate a candidate explanation or alternative hypothesis, but its epistemic status depends on interpretation, evidence, criticism, and practical evaluation by humans and institutions. AI may therefore support particular stages of inquiry without constituting Peircean inquiry itself.

For explainable AI, pragmaticism means that an explanation should be assessed not only for intelligibility or immediate usefulness but also for what it means in context, what follows from accepting it, what evidence supports it, and whether experience warrants revision. It thus connects meaning with responsible action.

#### 3.2.1 Fallibilism, critique, and the continuous development of knowledge

An equally important dimension of Peirce’s epistemology is fallibilism: the recognition that finite inquiry does not guarantee absolute certainty and that knowledge claims remain open to correction. Fallibilism should therefore not be reduced to the observation that AI systems can make mistakes. Its deeper significance concerns the status of knowledge claims and the practices through which those claims remain revisable. For Peirce, inquiry is self-correcting because beliefs can be challenged by experience, new evidence, and criticism within a community of investigators.

Peircean fallibilism has an important affinity with Karl Popper’s critical rationalism in rejecting infallible foundations and emphasizing provisional knowledge. Their emphases differ: Popper stresses conjecture, criticism, and falsification, whereas Peirce places fallibilism within a broader process of doubt, abduction, experience, experimentation, and communal inquiry. For XAI, this means explanations should support not only criticism but also alternative hypotheses, evidence examination, practical evaluation, and revision.

Abduction also requires qualification in the context of AI. The generation of a plausible hypothesis does not by itself establish abductive reasoning, since Peircean abduction involves a broader process of inquiry, evidence, and evaluation.

Accordingly, the framework treats pragmaticism and fallibilism as normative and epistemological resources, not descriptions of what AI literally does. Computational systems can generate outputs, incorporate feedback, communicate uncertainty, and update models, but these mechanisms do not automatically constitute Peircean inquiry, which involves judgments about evidence, relevance, criticism, and consequences within human and institutional communities.

For explainable and human-centred AI, explanations should remain open to questioning and correction. Uncertainty communication, alternative explanations, evidence checking, human review, and contestation are design and institutional conditions that keep AI-supported claims open to revision.

### 3.3 Semiotics and meaning construction

Peirce’s semiotic theory provides a philosophical framework for understanding how AI-generated representations participate in processes of meaning construction rather than treating AI itself as an autonomous producer of meaning. In Peirce’s triadic account, a sign relates to an object through an interpretant, and the significance of the sign develops through interpretation rather than residing in the representation alone (Peirce, CP 2.228). This distinction is particularly important for AI because a generated prediction, recommendation, classification, or textual response acquires practical significance only when it is interpreted in relation to an object, a context, and a purpose.

The concept of the interpretant is therefore central to the proposed account of explainability. An explanation is not epistemically complete merely because an AI system produces a representation of why an output was generated. The explanation must become interpretable to an audience within a particular context of inquiry. Different users may require different explanatory forms because their knowledge, purposes, responsibilities, and decision contexts differ. Human-centred explainability should consequently be understood as relational: an explanation is adequate not simply because information has been disclosed, but because it enables an appropriate interpretive relationship between the AI output, the relevant evidence, the user’s context, and the practical decision at stake.

In this framework, human-centredness does not mean that the preferences of the immediate user automatically determine what is appropriate. In consequential AI contexts, the relevant human perspective includes the direct user, persons affected by the system’s outputs, domain experts, and legitimate institutional or public stakeholders, according to the nature and risk of the application. This broader interpretation follows the logic of the community of inquiry: explanatory adequacy should remain open to perspectives that can identify errors, harms, missing evidence, or competing interpretations. Human-centredness therefore refers not simply to user convenience but to the capacity of AI systems and their surrounding institutions to support meaningful interpretation, participation, contestation, and accountable decision-making.

This triadic conception shifts attention from the manipulation of representations to the processes through which meaning is generated, interpreted, and revised in context (Colapietro, 2023). The question is therefore not only whether AI manipulates symbols successfully, but whether its outputs can participate in meaningful interpretation. Recent work on symbols in the AI era likewise distinguishes computational symbol manipulation from contextual meaning construction (Rong and Qing, 2026).

Within the proposed framework, AI-generated outputs can be analysed as signs within human processes of semiosis, whose significance depends on their interpretation in relation to context, evidence, and purpose. A medical prediction, legal recommendation, or generated text does not acquire practical meaning independently of the context in which it is evaluated and used. AI is consequently better understood as a technological mediator within human processes of meaning construction than as an autonomous producer of meaning.

Peirce’s semiotics also informs contemporary discussions of knowledge representation and XAI. Developments in natural-language processing, knowledge graphs, and neuro-symbolic AI seek richer semantic relationships and more interpretable results (Ekaputra, 2025; Platzer, 2024). From a Peircean perspective, these efforts address aspects of semiosis by enriching relations among signs, concepts, and contexts.

Peirce’s semiotics deepens explainability by distinguishing information disclosure from meaningful interpretation. An explanation becomes epistemically useful when it enables users to interpret and evaluate an output within a particular inquiry context (Miller, 2019; Arrieta et al., 2020). Explainability therefore includes both explanatory information and the human processes through which that information becomes meaningful.

Applying Peirce’s semiotics to AI does not imply consciousness, intentionality, or human-equivalent understanding. It provides a lens for examining how AI represents, transforms, and communicates information within socio-technical environments. These processes remain dependent on human interpretation, contextual judgment, and social practice (Viidalepp and Olteanu, 2025).

Across these domains, AI is best understood as a technological mediator rather than an autonomous knower. Its outputs may provide representations or recommendations, but their epistemic status depends on interpretive and institutional practices. In high-stakes settings, a Peircean approach therefore requires accessible explanations together with contextual interpretation, expert review, contestation, and correction.

### 3.4 Abduction and knowledge generation

Abduction represents one of Charles Sanders Peirce’s most significant contributions to the philosophy of logic and epistemology. It refers to the inferential process through which possible explanations are generated in response to unexpected observations or phenomena. Unlike deduction, which derives necessary conclusions from established premises, and induction, which develops generalizations from empirical observations, abduction involves proposing a plausible hypothesis that could explain an observed fact (Peirce, 1998). For Peirce, abduction constitutes the initial stage of scientific inquiry because it explains how new ideas and hypotheses emerge.

According to Peirce, scientific investigation does not begin with the mechanical application of existing rules but with the recognition of a surprising fact that requires explanation. When researchers encounter an unexpected phenomenon, they formulate a hypothesis that, if true, would make the phenomenon understandable. However, abductive reasoning does not establish certainty; rather, it produces possible explanations that must subsequently be examined through deduction, experimentation, and further inquiry (Peirce, 1998, pp. 400–402). Therefore, abduction represents a creative but fallible process through which knowledge develops.

Abduction is important because scientific inquiry requires new possibilities, not merely processing existing information. Peirce regarded it as the logic of discovery because it connects observation with hypothesis and unexplained phenomena with possible interpretations (Douven, 2021).

The philosophical status of abduction itself, however, requires qualification. Peirce’s characterization of abduction as a form of logical inference has been subject to criticism, particularly concerning the relationship between inferential structure, perception, creativity, and the formation of explanatory hypotheses. Hoffmann (1999), for example, argues that difficulties arise when abduction is treated as a fully specified logic of discovery without distinguishing its inferential elements from the perceptual dimensions involved in hypothesis formation. This debate is important for the application of Peircean abduction to AI because the production of a plausible output does not by itself establish the presence of abductive reasoning. The present framework therefore uses abduction in a deliberately qualified sense: AI systems may generate candidate hypotheses or explanations that are functionally analogous to the hypothesis-generating dimension of abduction, but they do not thereby instantiate the full epistemological process described by Peirce. Human inquiry remains necessary for determining whether a generated possibility is relevant, evidentially supported, practically adequate, and worthy of further investigation.

Abduction is closely connected to semiotics because interpreting a sign can involve proposing possible meanings that explain its relation to an object. It thus provides a logical mechanism through which semiosis can generate new interpretants and explanatory possibilities (Jappy, 2022).

This concept is relevant to generative AI, which can produce novel outputs and possible explanations. Yet statistical generation should not be equated with genuine abductive reasoning. AI can produce hypotheses based on learned patterns, but this does not by itself establish genuine abductive or explanatory reasoning.

The difference between human abduction and AI-generated responses lies in the broader context of inquiry. Human hypotheses are embedded in experience, intentionality, criticism, and communities of inquiry. AI generates outputs through training data, algorithms, and objectives. It may therefore simulate aspects of abduction without possessing the full interpretive and epistemic conditions of Peircean inquiry.

Peircean abduction is therefore most productive for AI research when used to analyze how AI-generated possibilities enter human knowledge construction rather than to attribute abductive agency to machines. It highlights both AI’s capacity to support hypothesis generation and the continuing human responsibility for interpretation, evidence, criticism, and epistemic evaluation.

### 3.5 Toward human-centred and explainable AI

The increasing integration of AI into social and professional domains has intensified the need for systems that are effective, interpretable, accountable, and aligned with human values. A Peircean perspective examines how computational outputs acquire meaning, are evaluated by people, and contribute to responsible knowledge practices. This aligns with responsible AI governance approaches emphasizing risk management, continuous evaluation, and accountability (Mökander et al., 2022).

Peirce’s philosophy supports this perspective because knowledge emerges through interpretation, criticism, and collective inquiry. AI outputs are not trustworthy merely because they are accurate; their significance depends on contextual evaluation by users and communities. Explainability, transparency, accountability, and oversight therefore become epistemological as well as ethical conditions. Semioethics similarly emphasizes the connection between meaning construction and responsibility (Dellino, 2025).

#### 3.5.1 Explainability and interpretation

Explainable AI (XAI) has developed into a diverse field that includes both technical approaches to model transparency and approaches concerned with how explanations are understood and used by people. Early XAI research emphasized interpretability, model transparency, feature attribution, and post-hoc explanations, but subsequent scholarship has increasingly questioned whether an explanation can be considered successful merely because it reveals information about a model. Interpretability and explainability are not conceptually identical, and neither can be understood independently of the audience, purpose, and context in which an explanation is received (Arrieta et al., 2020; Miller, 2019; Murdoch et al., 2019). In particular, human-centred XAI has emphasized that explanation should be designed around users’ needs, goals, expertise, and interaction with AI systems rather than treated exclusively as a property of the underlying model (Liao and Varshney, 2021). More broadly, pragmatist approaches to AI-related research emphasize the importance of context, design, and practical performance evaluation when assessing AI-supported systems (Love, 2024).

Trust should also be distinguished from mere acceptance of an AI output. Within the proposed framework, trust is a provisional and revisable practical attitude grounded in evidence, performance, transparency, accountability, and the availability of correction and contestation mechanisms. An explanation may contribute to justified reliance when it enables users to understand relevant reasons and limitations, but explanation alone does not warrant trust. Indeed, Peircean fallibilism implies that responsible trust must remain compatible with the possibility of error and revision. Trustworthy AI is therefore not AI that users simply believe; it is AI embedded in practices that allow reliance to be informed, monitored, questioned, and corrected.

This development is important because contemporary XAI already recognizes the role of human interpretation. Human-centred approaches examine how explanations support understanding, decision-making, and interaction, while HCI research shows that explanations contribute to users’ mental models (Poursabzi-Sangdeh et al., 2021; Sokol and Flach, 2021). The Peircean contribution is to explain why interpretation is constitutive of explanatory significance and why explanations should remain open to evaluation and revision.

This distinction clarifies interpretability and explainability. Intrinsically interpretable models expose aspects of their operation through their structure, whereas post-hoc methods provide additional representations of otherwise opaque models (Rudin, 2019; Marcinkevičs and Vogt, 2023). Neither guarantees understanding or justified reliance. Explanatory adequacy varies with expertise, purpose, stakes, and context, making explainability relational rather than purely technical.

Peircean explainability therefore extends rather than rejects contemporary XAI. Technical methods remain necessary for revealing model behaviour, influential features, causal relations, or counterfactual alternatives. Their value, however, depends on whether users can interpret the information in relation to their questions, evidence, and practical purposes. Such methods are thus resources within inquiry rather than self-validating knowledge.

#### 3.5.2 Transparency and accountability

Transparency can contribute to justified trust, but it is not sufficient or necessary in every context. From a Peircean perspective, information has epistemic value when it enables users and institutions to investigate outputs, assess evidence and limitations, and revise judgments. Transparency therefore matters insofar as it supports inquiry and accountability rather than disclosure for its own sake (Blanchard et al., 2025).

AI systems increasingly influence decisions in areas such as healthcare, education, justice, governance, and scientific research. Consequently, questions of responsibility cannot be separated from the operation of these systems. If AI outputs affect human decisions, mechanisms must exist to examine, question, and revise these outputs when necessary. This principle corresponds with Peirce’s conception of inquiry as an open and self-correcting process in which knowledge remains subject to criticism and improvement (Hookway, 2000; Misak, 2004).

Accountability in AI requires a clear relationship between computational systems and human responsibility. Semioethical approaches likewise connect interpretation with responsibility (Zengiaro, 2025). AI may provide analytical support or recommendations, but interpretation and ethical evaluation remain human responsibilities. Accountable AI therefore depends on institutional practices of review, justification, and correction.

#### 3.5.3 Human oversight and responsible AI

A central implication of Peirce’s philosophy for AI is the importance of maintaining human participation in processes of knowledge formation and decision-making. Although contemporary AI systems demonstrate increasing capacities for prediction and generation, they do not operate as independent epistemic agents. Within the proposed framework, the epistemic significance of AI outputs is constituted through human interpretation, contextual understanding, and social evaluation.

Peirce’s concept of the community of inquiry provides a useful basis for responsible AI. Knowledge develops through criticism, dialogue, and communal inquiry; similarly, AI should support human inquiry rather than replace human judgment. Oversight is therefore not merely a technical safeguard but a condition for responsible knowledge formation.

The community of inquiry should not, however, be interpreted as an automatically egalitarian process. In real AI deployments, users, affected individuals, developers, institutions, and regulators may possess unequal access to information, technical expertise, and decision-making authority. A meaningful community of inquiry therefore requires institutional mechanisms that make relevant evidence accessible, provide channels for questioning and appeal, and give affected stakeholders a genuine opportunity to challenge consequential AI outputs. The Peircean ideal is consequently normative rather than descriptive: it provides a criterion for evaluating whether an AI decision environment remains sufficiently open to criticism, participation, and correction despite existing asymmetries of power.

This conception also converges with the emerging literature on contestable AI, which emphasizes that affected individuals and human controllers should be able to question automated decisions, request human review, provide additional information, and obtain reconsideration where appropriate. Contestability therefore involves more than placing a human somewhere in the decision loop; it requires procedural opportunities for dialogue, intervention, and revision (Almada, 2019; Vaccaro et al., 2019; Lyons et al., 2021). Recent work has further conceptualized contestability as a design property that should support explanations, human intervention, and scrutiny throughout the AI lifecycle. From a Peircean perspective, these mechanisms can be interpreted as institutional conditions for a community of inquiry: they create opportunities for claims generated or supported by AI systems to be questioned, examined, challenged, and revised. The contribution of the Peircean framework is therefore to connect contestability not only with procedural fairness or governance, but also with the epistemic conditions required for fallible knowledge to remain open to correction.

Contemporary governance approaches likewise emphasize human-centred responsibility and oversight. The community of inquiry reframes governance as collective evaluation, interpretation, and correction rather than merely technical control (Emery-Xu et al., 2025; Mökander et al., 2022).

This approach rejects the view of AI as an autonomous producer of knowledge. AI can extend analytical capacities by processing information and generating possibilities, while humans remain responsible for meaning, relevance, fairness, and consequences.

Accordingly, responsible AI requires integrating technological innovation with philosophical reflection on human agency, interpretation, and accountability. A Peircean framework highlights that the value of AI lies not in replacing human cognition but in supporting more effective and reflective processes of inquiry.

#### 3.5.4 Social applications of the Peircean framework for AI

A Peircean understanding of AI is especially relevant in domains where interpretation and judgment matter. In healthcare, AI-assisted diagnosis should be integrated with clinical expertise and contextual knowledge. In education, recommendations should support teacher and student interpretation rather than replace it. In justice and public administration, AI should support deliberation while remaining subject to review concerning fairness, legitimacy, and accountability.

In healthcare, for example, AI-assisted diagnostic systems can be understood as interpretive tools in which medical data function as signs requiring evaluation by healthcare professionals. The meaning of an AI-generated diagnosis does not emerge from computational analysis alone but through integration with clinical expertise, contextual knowledge, and professional judgment.

Similarly, in education, AI systems that provide personalized learning recommendations or feedback should be evaluated according to their ability to support meaningful learning interactions. Their effectiveness depends not only on accuracy but also on whether teachers and students can interpret and critically engage with their outputs.

In justice and public administration, AI applications raise additional questions concerning fairness, legitimacy, and accountability. Because decisions in these fields involve social values and ethical considerations, AI systems should support human deliberation rather than replace institutional judgment. A Peircean approach emphasizes that responsible decision-making requires continuous evaluation within communities of inquiry.

Across these domains, AI is a socio-technical participant in meaning mediation rather than an autonomous knower. Work on AI and Theory of Mind similarly cautions against equating computational modelling with genuine intentionality or consciousness (Nebreda et al., 2023). The Peircean framework therefore combines technical capability with meaning, accountability, and collective inquiry.

Accordingly, a Peircean approach to human-centred and explainable AI integrates technical capabilities with philosophical considerations of meaning, transparency, accountability, and social responsibility. It reinforces the view that the future of AI depends not only on developing more powerful systems but also on ensuring that these systems remain embedded within human-centred processes of understanding and collective inquiry.

## 4 Conceptual framework: from Peircean concepts to XAI design and governance

The philosophical reconstruction described above provides the basis for the proposed framework. Rather than treating Peircean concepts as direct computational equivalents, the framework identifies their epistemic functions and examines how these functions can inform the design, interpretation, evaluation, and governance of explainable and human-centred AI. In this sense, the framework operates at the level of the relationship between AI-generated outputs and the human practices through which those outputs are interpreted, questioned, evaluated, and potentially revised. It therefore derives principles for explanation, interaction, evaluation, and governance from the epistemic functions of semiosis, abduction, inquiry, fallibilism, pragmaticism, and the community of inquiry.

The mapping presented in Table 1 should not be understood as a claim that AI systems literally instantiate Peircean forms of reasoning. Rather, it identifies the epistemic and normative functions that can be supported when AI-generated outputs are incorporated into human practices of explanation and inquiry. This distinction is essential: the computational system produces an output, but the epistemic status of that output is established through interpretation, evidential assessment, criticism, and practical evaluation. The framework therefore assigns a differentiated role to AI and human agents. AI systems may generate representations, candidate explanations, alternatives, or uncertainty information, whereas human users and institutions remain responsible for interpreting these resources, assessing their evidential adequacy, contesting them when necessary, and determining whether they are appropriate for action.

**Table 1:** Peircean epistemic functions and their implications for XAI design and governance.

Peircean concept	Epistemic function	XAI function	Design and governance implication	Human role
Semiosis	Meaning emerges through the interpretation of signs in context	Provide explanations that connect outputs with relevant context, evidence, and user goals	Context-sensitive and audience-appropriate explanations	Interpret the significance and relevance of the explanation
Abduction	Generate plausible hypotheses without establishing their truth	Present possible reasons, hypotheses, or alternative explanations	Distinguish hypotheses from established conclusions and communicate evidential status	Assess plausibility and compare alternatives with available evidence
Inquiry	Test, question, criticize, and revise beliefs	Support interactive questioning, evidence checking, and explanation revision	Enable iterative explanation, feedback, and correction	Question outputs, request clarification, and revise judgments
Fallibilism	Treat knowledge claims as potentially incomplete or erroneous	Communicate uncertainty, limitations, and possible errors	Include uncertainty disclosure, error reporting, and correction mechanisms	Remain open to revising judgments when new evidence emerges
Pragmaticism	Evaluate meaning through conceivable practical consequences	Relate explanations to concrete tasks, decisions, and outcomes	Assess explanatory usefulness in the context of actual use rather than abstract transparency alone	Evaluate whether the explanation supports appropriate action and understanding
Community of Inquiry	Establish knowledge through collective criticism, dialogue, and communal inquiry	Enable review, contestation, stakeholder participation, and institutional oversight	Provide mechanisms for human review, appeal, contestability, and collective scrutiny and correction	Participate in deliberation, challenge decisions, and exercise accountability

The six dimensions are also mutually reinforcing rather than sequential in a strictly linear sense. An interpreted output may generate a new question; a question may lead to additional evidence; new evidence may undermine an earlier hypothesis; and revision may produce a new interpretive context. Explainability is therefore conceived as an iterative process of semiosis and inquiry in which interpretation, hypothesis formation, evaluation, and correction can recur throughout the interaction between humans and AI. This iterative structure is particularly important for human-centred AI because it prevents explainability from being reduced to a one-time disclosure of model information.

Figure 1 presents the proposed framework as an iterative rather than linear process. AI-generated outputs enter human practices as signs requiring interpretation; interpretation may generate abductive possibilities, which are then subjected to inquiry through questioning, evidence checking, and practical evaluation. When relevant stakeholders identify uncertainty, error, or disagreement, the output or explanation can be contested and revised, returning the process to further interpretation and inquiry. Fallibilism operates across the entire cycle by keeping knowledge claims open to correction, while the community of inquiry provides the social and institutional conditions for responsible evaluation. The figure therefore emphasizes that Peircean explainability is not completed when an explanation is generated; it is sustained through continuing processes of interpretation, evaluation, contestation, and revision.

**Figure 1:**
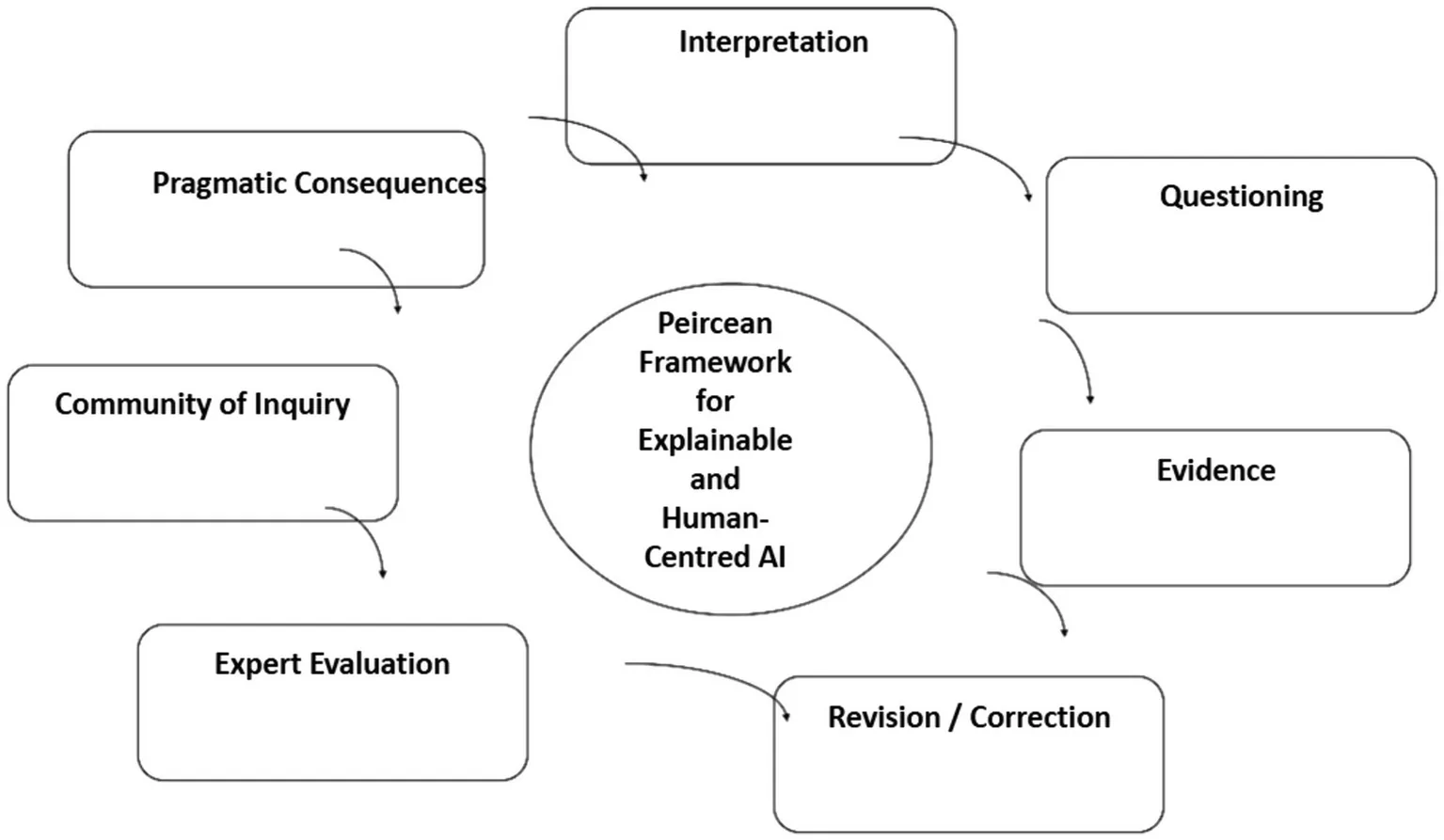
Iterative Peircean framework for explainable and human-centred AI.

## 5 Applying the Peircean framework to explainable AI

The following example illustrates how the proposed framework can complement existing XAI practices by treating explanation as an iterative process of interpretation and inquiry. The example is conceptual rather than empirical: it does not evaluate ChatGPT’s performance, measure user comprehension, or claim that the system itself performs Peircean reasoning. Its purpose is to demonstrate how an AI-generated explanation can be incorporated into a human-centred epistemic process.

Conventional XAI often focuses on model behaviour, statistical learning, or feature attribution (Arrieta et al., 2020; Miller, 2019). The Peircean perspective adds the interpretive process through which outputs become meaningful for users (Jappy, 2022; Viidalepp and Olteanu, 2025).

Within this framework, a user’s prompt can be treated analytically as a sign within a broader process of semiosis. The AI system processes the prompt through learned statistical patterns and generates a response, which can in turn function as a sign when it is interpreted by a human user in relation to an object, context, and purpose. Within this framework, explanation is provisionally realized through the relationship between an AI output and its human interpretation (Colapietro, 2023).

### 5.1 Worked example: from AI explanation to human inquiry

Consider a user who asks ChatGPT to explain why a particular student might be at risk of poor academic performance. Suppose the system responds that the risk is associated primarily with low attendance and limited engagement with course materials. Under a conventional understanding of explainability, the response might be treated as an explanation if the system provides a plausible account of the factors associated with the prediction. The Peircean framework, however, treats the response as the beginning rather than the completion of explanation.

First, semiosis requires the user to interpret the output in relation to the particular student, educational context, and purpose of the decision. Second, abduction treats the proposed factors as candidate hypotheses rather than established causes. The user may therefore ask whether other explanations are possible, such as difficulties with access to learning resources, workload, or circumstances not represented in the available data. Third, inquiry requires these possibilities to be examined against relevant evidence rather than accepted because they were generated by the AI system. Fourth, fallibilism requires the user to recognize that the original explanation may be incomplete or mistaken. Fifth, pragmaticism directs attention to whether the explanation helps educators decide what form of support would actually be appropriate. Finally, the community of inquiry requires the relevant human participants, such as teachers, administrators, or other authorized stakeholders, to review the interpretation and contest it when necessary.

The example demonstrates the framework’s distinctive role: ChatGPT generates a possible explanation but does not establish its validity or practical appropriateness. The process remains provisional through human interpretation, evidence-based evaluation, dialogue, and revision. If new evidence changes the assessment, inquiry reopens (see Table 2).

**Table 2:** Peircean dimensions in the worked XAI example.

Peircean dimension	Function in the example	Human contribution
Semiosis	Interpreting the AI explanation in context	Determine relevance and significance
Abduction	Generating alternative explanations	Compare plausible hypotheses
Inquiry	Testing explanations against evidence	Question and verify claims
Fallibilism	Maintaining openness to error	Revise conclusions when warranted
Pragmaticism	Assessing practical consequences	Evaluate whether the explanation supports appropriate action
Community of Inquiry	Collective review and contestation	Deliberate, challenge, and authorize revision

Abduction clarifies why AI-generated explanations should be treated as hypotheses rather than conclusions. They require comparison with evidence and critical reflection before being accepted as reliable knowledge (Peirce, 1998; Douven, 2021). Inquiry then permits users to reformulate prompts, compare responses, consult external sources, and revise judgments. Human oversight connects these processes to trustworthy AI because epistemic validity and ethical acceptability are not determined by the system alone (Mökander et al., 2022; Emery-Xu et al., 2025).

## 6 Discussion: Peirce’s pragmatic semiotics and the future of explainable AI

The analysis shows that Peirce’s philosophy provides more than a historical interpretation of pragmatism: it offers a philosophical framework for understanding AI as an interpretive and epistemic practice. Contemporary XAI includes interpretable models, post-hoc explanations, causal and counterfactual methods, and human-centred approaches. The remaining philosophical question is how these explanatory resources acquire epistemic significance within particular practices of inquiry.

From a Peircean perspective, explainability is a process of semiosis: AI outputs function as signs whose significance emerges through interpretation in context. Explanation therefore includes not only information about model behaviour but also how users evaluate evidence and integrate outputs into practical reasoning.

### 6.1 Comparison with mainstream explainable AI approaches

The proposed framework should not be interpreted as an alternative to contemporary XAI techniques but as a philosophical layer that clarifies their epistemic role. Contemporary XAI encompasses a diverse range of approaches, including intrinsically interpretable models, post-hoc feature-attribution methods, example-based explanations, causal and counterfactual explanations, and increasingly human-centred approaches concerned with users, context, interaction, and mental-model formation. These approaches address different aspects of the explainability problem and cannot be reduced to a single technical paradigm.

The distinctive contribution lies in asking how explanatory resources function within inquiry. Feature attribution, causal and counterfactual explanations, and human-centred methods provide different forms of information; the Peircean perspective asks how that information is interpreted, assessed against evidence, challenged, and revised. It thus connects technical explanation with understanding, justified reliance, contestability, and responsible decision-making.

The novelty is philosophical and normative rather than algorithmic or empirical. The framework reconstructs explanation as situated inquiry and identifies conditions for meaningful, revisable, and contestable explanations. It connects interpretation, hypothesis formation, uncertainty, practical consequences, human oversight, and collective contestation within one epistemic model.

Figure 2 illustrates how established XAI techniques can be situated within the broader Peircean model of epistemic inquiry. Rather than replacing these techniques, the framework interprets them as resources that support different functions within a continuing process of interpretation, evaluation, contestation, and revision.

**Figure 2:**
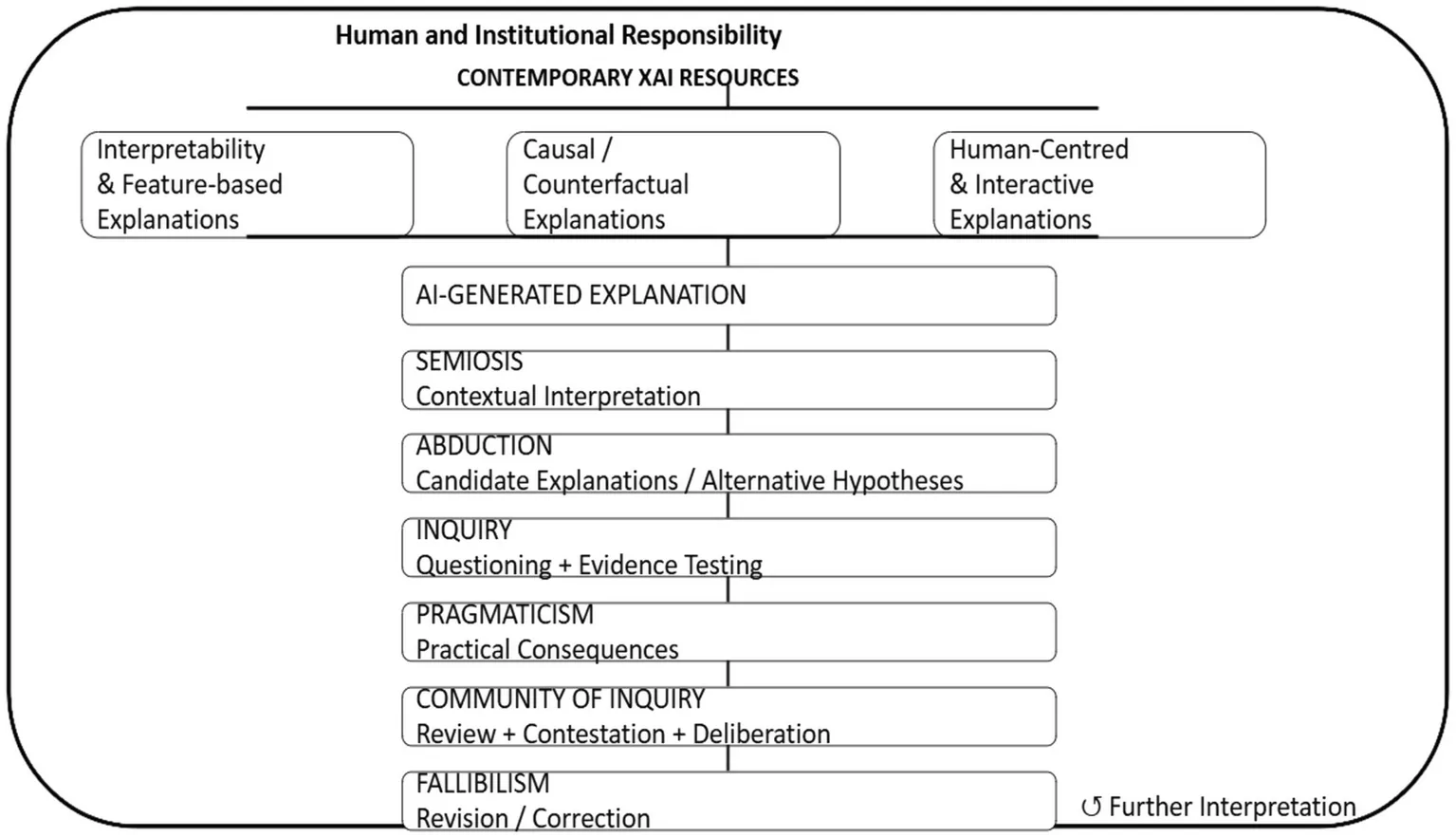
From explanatory techniques to Peircean epistemic inquiry.

The analysis also demonstrates that Peirce’s concepts of inquiry, fallibilism, and the community of inquiry provide important philosophical resources for understanding trustworthy and human-centred AI. Many contemporary AI deployments involve adaptation, updating, or interaction with changing environments. Although these processes may resemble aspects of Peirce’s conception of inquiry, they should not be interpreted as evidence that AI possesses genuine understanding or autonomous cognition. Instead, intelligent systems remain epistemic instruments whose outputs remain subject to human interpretation, criticism, and revision.

This perspective has important implications for AI governance. Current governance frameworks frequently emphasize transparency, accountability, and human oversight as regulatory principles. A Peircean framework deepens these principles by explaining why they are philosophically necessary. Human oversight is not merely a safeguard against technical failure; it is an epistemological requirement because the meaning and validity of AI-generated outputs emerge only through interpretive practices conducted within communities of inquiry. Trustworthy AI therefore depends not only on computational reliability but also on the social processes through which explanations are evaluated, questioned, and continuously revised.

## 7 Conclusion

This study has argued that Charles Sanders Peirce’s pragmatic semiotics provides a coherent framework for rethinking AI beyond purely computational perspectives. By integrating semiosis, abduction, inquiry, fallibilism, pragmaticism, and the community of inquiry, it shows that AI outputs acquire epistemic significance through human interpretation, contextual judgment, and collective evaluation. Explainability is therefore a dynamic process of meaning construction within inquiry.

The framework connects Peircean semiotics with contemporary debates on XAI, trustworthy AI, and HCAI. It does not replace computational methods; it identifies epistemological conditions under which their outputs can become meaningful, intelligible, revisable, and socially accountable.

The analysis further shows that human oversight, transparency, accountability, and critical evaluation can be understood as epistemological requirements, not only ethical principles. Trustworthy AI therefore depends on practices that allow people to evaluate, contest, and revise AI-supported claims.

Future interdisciplinary research can apply the framework to generative AI, clinical decision support, education, and public-sector systems to examine how Peircean principles inform explanation, evaluation, and governance.

### 7.1 Limitations

The present study is conceptual rather than empirical and reconstructs a philosophical framework rather than evaluating specific AI systems or explanation techniques. It has not been empirically validated through case studies, user experiments, or comparative assessments, and it focuses on Peirce rather than systematically comparing alternative philosophical traditions. A further limitation is that the proposed mapping from Peircean concepts to XAI design and governance remains theoretically specified; future research must determine whether it improves measurable outcomes such as understanding, appropriate reliance, contestability, or decision quality.

### 7.2 Future research

Future research may extend the proposed framework in several directions. First, empirical studies could investigate whether explanations designed according to Peircean principles improve users’ understanding, trust, and decision-making compared with conventional XAI techniques. Second, comparative research may examine how the proposed framework relates to other philosophical approaches to AI explainability, including hermeneutic, phenomenological, or virtue-epistemological perspectives. Third, interdisciplinary work could operationalize Peirce’s concepts within practical AI domains such as healthcare, legal decision support, education, and public administration to evaluate their usefulness for the design of human-centred explainable systems. Finally, future studies may explore how Peirce’s notion of the community of inquiry can inform collaborative AI governance and participatory approaches to responsible AI.

## Data Availability

The original contributions presented in the study are included in the article/supplementary material, further inquiries can be directed to the corresponding author.
